# The Impact of Movements and Animal Density on Continental Scale Cattle Disease Outbreaks in the United States

**DOI:** 10.1371/journal.pone.0091724

**Published:** 2014-03-26

**Authors:** Michael G. Buhnerkempe, Michael J. Tildesley, Tom Lindström, Daniel A. Grear, Katie Portacci, Ryan S. Miller, Jason E. Lombard, Marleen Werkman, Matt J. Keeling, Uno Wennergren, Colleen T. Webb

**Affiliations:** 1 Department of Biology, Colorado State University, Fort Collins, Colorado, United States of America; 2 Center for Complexity Science, Mathematics Institute, University of Warwick, Coventry, United Kingdom; 3 Department of Physics, Chemistry, and Biology, Linköping University, Linköping, Sweden; 4 United States Department of Agriculture, Animal and Plant Health Inspection Service, Centers for Epidemiology and Animal Health, Fort Collins, Colorado, United States of America; Northeastern University, United States of America

## Abstract

Globalization has increased the potential for the introduction and spread of novel pathogens over large spatial scales necessitating continental-scale disease models to guide emergency preparedness. Livestock disease spread models, such as those for the 2001 foot-and-mouth disease (FMD) epidemic in the United Kingdom, represent some of the best case studies of large-scale disease spread. However, generalization of these models to explore disease outcomes in other systems, such as the United States’s cattle industry, has been hampered by differences in system size and complexity and the absence of suitable livestock movement data. Here, a unique database of US cattle shipments allows estimation of synthetic movement networks that inform a near-continental scale disease model of a potential FMD-like (i.e., rapidly spreading) epidemic in US cattle. The largest epidemics may affect over one-third of the US and 120,000 cattle premises, but cattle movement restrictions from infected counties, as opposed to national movement moratoriums, are found to effectively contain outbreaks. Slow detection or weak compliance may necessitate more severe state-level bans for similar control. Such results highlight the role of large-scale disease models in emergency preparedness, particularly for systems lacking comprehensive movement and outbreak data, and the need to rapidly implement multi-scale contingency plans during a potential US outbreak.

## Introduction

Outbreaks of rapidly spreading infections in populations of livestock around the world can have far reaching economic impacts. Direct costs of the 1997 FMD epidemic in Taiwan were estimated at $387.6 million, while the total cost was determined to be closer to $1.6 billion [1]. Similarly, the 2001 epidemic in the UK was estimated to have cost £3.1 billion to agriculture with similar, associated losses to tourism [2]. With a cattle population that is nearly an order of magnitude larger than that in the UK, the potential impacts of a rapidly spreading disease like FMD on the US economy are staggering. Mechanistic models of the spread of an FMD-like disease in the US can help to mitigate these potential costs by providing robust explorations of the effects of scale and regionalization on potential surveillance and control measures. In particular, retrospective models of the 2001 UK outbreak provide insights on the influence of premises and animal densities on spatial dynamics of transmission [3]–[8] and the utility of detailed animal movement information in prediction of long-range disease spread [9]–[14].

Long-distance transmission is of particular concern when studying outbreaks at a larger spatial scale, and although mechanisms (e.g., tagging of certain animals) exist in the US to support animal tracing during an outbreak, these data are not readily available. Most publicly available information on livestock distribution in the US is aggregated at the county level owing to confidentiality concerns [15], and even the best source of national animal movement data (i.e., Interstate Certificates of Veterinary Inspection; ICVIs) is incomplete owing to reporting requirements designed to ensure compliance with state and federal animal health import requirements as opposed to comprehensive movement tracking (see Materials and Methods). Previous characterizations of US cattle movements were therefore based on coarse summary data describing the volume of cattle moving between a subset of states [16], and existing models of disease spread in the US cattle industry lack an explicit, data driven movement network encompassing the entire industry [17]–[19]. In all, US livestock disease models face three inherent challenges not encountered in previous livestock disease models: 1) incomplete cattle movement information to characterize long-distance spread; 2) spatially aggregated premises location data prohibiting models of distance-based premises-to-premises spread; and 3) lack of outbreak data to parameterize epidemiological rates. We address the first challenge using a unique sample of ICVI records that, when incorporated into a spatially explicit movement kernel parameterized through Bayesian inference, allows us to create the first comprehensive cattle movement network model for the US. To address challenge two, a novel county-level metapopulation model is used to capture disease spread and assess control strategies. The parsimony of this model allows for extensive sensitivity analyses of epidemiological parameters to explore the impacts of challenge three (see Section E in Text S1) and also allows for the potential to fit the model during the early stages of a US outbreak.

## Materials and Methods

### ICVI Data

When livestock cross state lines, they are usually required to be accompanied by an Interstate Certificate of Veterinary Inspection (ICVI). A notable exception to this ICVI import requirement is cattle going directly to slaughter, although these movements are less important for transmission dynamics. ICVIs are official documents issued by a veterinarian accredited by USDA Animal and Plant Health Inspection Service-Veterinary Services who certifies animal health during an inspection prior to shipment. Additional copies of the ICVI are sent for approval and storage to the state veterinarian’s office in both the state where the shipment originated and the state of destination. Because ICVIs are issued by individual states, forms differ from state to state. However, all ICVIs list the origin and destination address for the livestock shipment providing a useful source of data on interstate cattle movements. In addition, ICVIs contain varying quality information on the following: shipment date, purpose (e.g., feeding, breeding, show/exhibition), production type (i.e., beef or dairy), breed, sex, and age [20].

To facilitate sampling, we requested that state veterinarians’ offices sample 2009 export ICVIs (see Section A in Text S1). ICVIs were sampled systematically by taking every 10^th^ cattle record. In most cases states either sent the 10% sample or sent all of their 2009 export ICVIs, which were subsequently sampled using the same design (see Section A in Text S1 for exceptions). Our ICVI sample contains 19234 non-slaughter movement records from 49 states and 2433 counties with New Jersey being the only state that did not provide data.

### ICVI Network

Network models consist of a set of nodes representing the individual units of study and a set of edges that describe interactions between nodes. In our case, nodes are defined as either counties or states in the US, and edges indicate that nodes are connected by a shipment of cattle. Edges in the model are directed (i.e., shipments have a defined start and end point) and weighted by the total number of shipments that move between nodes. Movement between nodes can now be described by paths, or any sequence of steps that can be taken to get from one node to another. We calculated several statistics that capture the overall structure of the US cattle movement network, including the diameter (i.e., the longest, shortest path length between any two nodes using unweighted edges) and the giant strongly connected component (i.e., GSCC, the largest set of nodes for which all pairs are reachable by a path in either direction). We also calculated a node’s in-degree (i.e., the total number of imports to a node) and out-degree (i.e., the total number of exports from a node). We calculated the network statistics using the igraph package [21] for R statistical software [22].

### Bayesian Networks

Due to the partial observation of the cattle movement network, some method of estimating the total number of movements between counties is required to simulate disease spread on this network. Contact heterogeneities induced by spatial clustering as well as industry structure are known to have important consequences for disease spread dynamics [23] and hence need to addressed in this estimation. We therefore used a spatially explicit kernel method based on Bayesian inference that makes three different assumptions about the cattle movement in the US system: 1) the probability of movement between counties decreases with distance; 2) the probability of movement is dependent on the number of premises in a county; and 3) cattle industry infrastructure and production are highly variable between states influencing the number of shipments sent and received [24]. The model, parameter estimation and validation are comprehensively described in Lindström et al. [24], or see Section C in Text S1 for a brief description).

### Disease Model

A novel, stochastic metapopulation disease model [25], [26] was developed that operates at the county scale and incorporates both local density-dependent spread and movement-based spread (see Table 1) along with culling of identified infected premises (IP). The disease simulations are based on a conceptualization where the premises is the basic unit of infection (see Section D in Text S1 for a complete description); that is, all animals within a premises become rapidly infected such that the entire premises can be classified as Susceptible, Exposed, Infectious or Removed. Premises-to-premises transmission occurs by two routes. First, local, non-movement contacts can result in aerosol, fence-line contact, or fomite transmission that are captured by a density- and distance-dependent spread process that is spatially localized within a county and between adjacent counties (see Table 1 and Figure S1). Second, long range movement transmission due to the shipping of animals between premises can occur between any two counties in the US (Table 1). However, while we consider transmission at the individual premises scale, data are only available at the county scale. This county-based aggregation leads to a stochastic metapopulation model whereby the population is divided geographically into a number of discrete patches, which we define as US counties [27]–[29].

**Table 1:** Disease transmission routes in the model.

	Movement spread*	Non-movement spread	
		*Within-county*	*Local cross-border*
**Cause**	Animal Shipments	Aerosol, fence-line contact, or fomite transmission	Aerosol, fence-line contact, or fomite transmission
**Spatial Scale**	All counties in the US	Premises within an infected county	All neighboring counties
**Assumptions**	1) Premises density-dependent; 2)Spatially explicit†; 3)Differs by state and production type	1) Premises density-dependent; 2)Premises size dependent	1) Premises density-dependent‡; 2)Premises size dependent‡; 3) Spatially implicit§
**Informed by or data from**	1) ICVI records; 2) Number of premises by county and production type¶; 3) State cattle inflows [38]	1) 2001 UK FMD outbreak [39]; 2) US premises density and size distributions¶	1) 2001 UK FMD outbreak [39]; 2) US premises density and size distributions ¶; 3) Shared county border length
**Parameter Uncertainty**	Estimated through Bayesian inference and incorporated in the simulations via multiple realizations of shipment networks.	Broad parameter ranges explored in a sensitivity analysis||.	Broad parameter ranges explored in a sensitivity analysis||.

*See Section C in Text S1 and Lindström et al. [24].

† Based on county centroids.

‡ In both the focal and neighboring counties.

§ Based on randomly distributed premises in the focal and neighboring counties.

¶ See Section B in Text S1 and NASS census data [15].

|| See Section E in Text S1.

Within each county, the population is considered to be well-mixed, consistent with the metapopulation formulation. However, in keeping with our conceptualization of the processes, local contacts are implicitly spatial and therefore depend on local density. We use the total number of cattle premises in each county from the 2007 Census of Agriculture conducted by the USDA National Agricultural Statistics Service (NASS) data as the base population in each county [15] and work with the number of premises of each epidemiological classification in each county (Susceptible, Exposed, Infectious, or Removed). At the start of the simulation all premises are assumed to be susceptible. These become infected through estimates of localized within- or between-county transmission, or movement-based transmission and move into the exposed class. Unless stated otherwise, we assume disease parameters for a rapidly spreading FMD-like disease. The mean exposed (latent) period is 5 days after which the premises becomes infectious and actively transmits (see Table 2). The mean delay from a premises becoming infectious and that premises being removed is 7 days (see Table 2), in line with previous work for time to depopulation in the 2001 UK epidemic [3], [6]. A thorough sensitivity analysis of transmission parameters was also performed (see Section E in Text S1 and Table 2).

**Table 2:** Disease simulation model parameters.

Type	Parameter	Value	Range	Description
Transmission	*β*	0.0003508*	[2×10^−5^, 4×10^−2^]	Transmission rate between cattle on different premises
	*α*	4.6†	[2.1, 6]	Shape of the local, non-movement spatial kernel
	*θ*	1.6‡	[1], [6]	Scale of the local, non-movement spatial kernel
	*p*	0.414†	[0, 1]	Non-linear scaling of the effect of premises size (i.e., number of cattle) on susceptibility to infection
	*q*	0.424†	[0, 1]	Non-linear scaling of the effect of premises size (i.e., number of cattle) on transmission of infection
Control	*ε*	100%†	[50%,100%]	Percentage of movements to/from an area that are stopped by a movement ban
	*λ*	7§	7, 14, 21	The delay between a premises becoming infected and subsequently being identified and removed, which triggers movement bans
Other	*σ*	5§	NA¶	The latent period; amount of time between a premises being exposed to infection and becoming infectious

^*^Units in Premises (days) ^−1^.

† Unit-less parameter.

‡ Units in kilometers.

§ Units in days.

¶ Sensitivity analysis was not performed on this parameter.

When studying the effect of movement restrictions, we assumed that any movement ban was 100% effective, in that all movements to and from the movement ban area would stop once introduced, and that a movement ban was introduced on the same day that the first infectious premises in a region was removed (i.e. a 7 day delay from a premises becoming infectious). We also explored the effect of movement ban effectiveness of stopping 100%, 90%, 75% and 50% of movements, coupled with a time delay to implementation of the movement ban from the first premises becoming infectious of 7 days, 14 days and 21 days.

For all of the analyses described in this paper, 100 epidemics were seeded in each of the 3109 counties in turn to allow for an investigation of the impact of the precise location of the source of the outbreak upon the spread of disease. For each epidemic, we measured the epidemic extent (i.e., number of counties infected) and the epidemic size (i.e., number of farms infected). Across all simulations, we also measured each county’s infection risk (i.e., the proportion of epidemics a county is affected by when seeding infection in each of the 3109 counties). Each of the 100 simulations in a given county utilized a different realization (as sampled from the posterior predictive distribution of movements) of the Bayesian movement kernel described above. The model was programmed in FORTRAN.

## Results and Discussion

### Cattle Movement Networks

**Figure 1:**
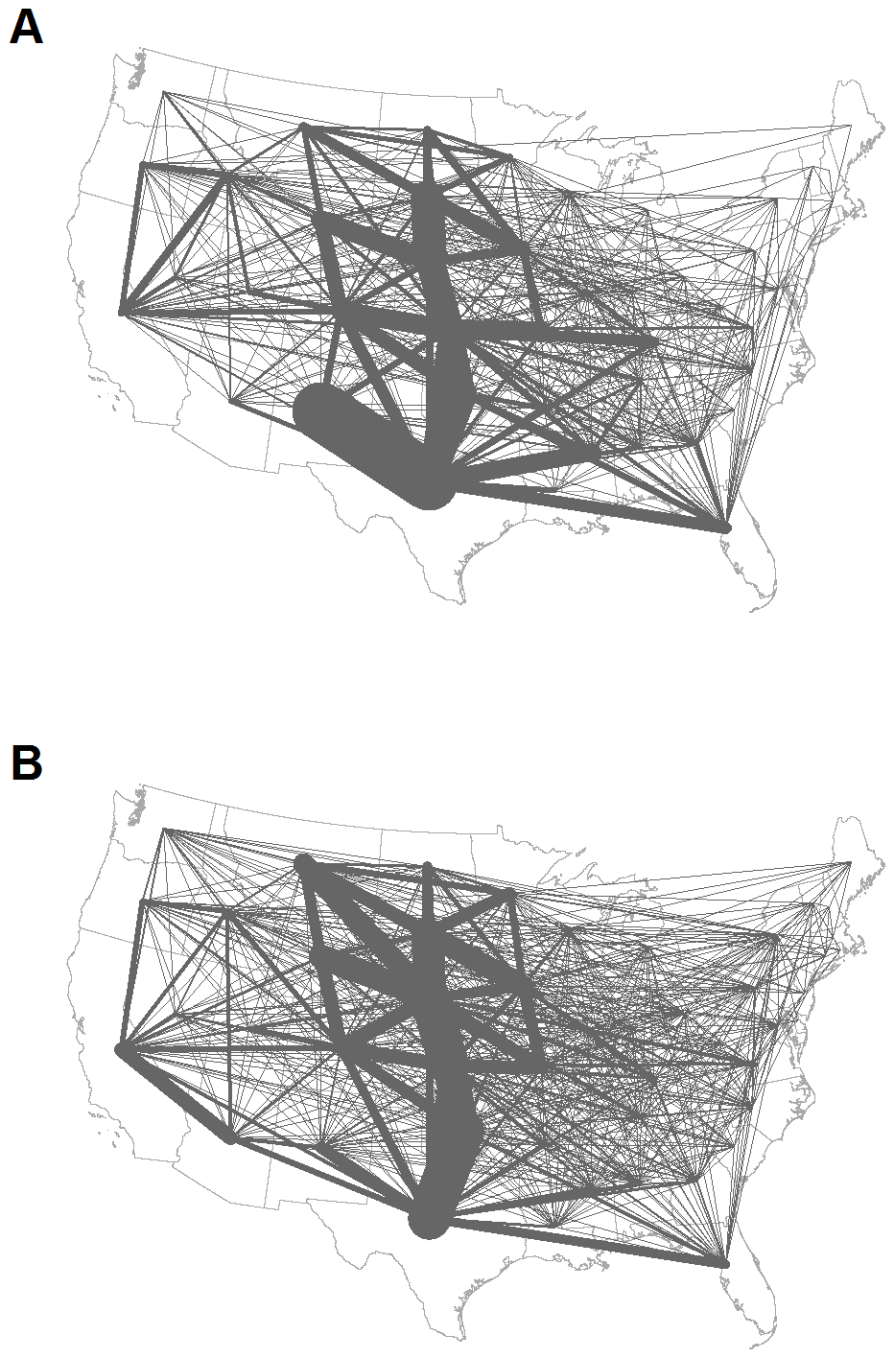
State-to-state cattle flows. Given for the (A) ERS ICVI summary data [16] and (B) 10% sample of paper ICVIs.

Movement patterns are dominated by movements to and from the Central Plains states (Figure 1). These states boast the majority of US feeder cattle, reflecting the large percentage of sampled ICVIs filed for feeding purposes (44.8%), although breeding (16.8%) and show/exhibition/rodeo (7.2%) movements are also common. Shipments were generally small with 81.7% containing fewer than 100 head of cattle and 38.2% containing fewer than 10, which, in general, matches the prevalence of US premises with fewer than 100 head of cattle (90.4% of beef premises [30]; 76.7% of dairy premises [31]). These general trends in the sampled ICVIs are consistent with a large central feeding system that amasses cattle from numerous relatively small holdings [30]-[32]. Although this database is the first of its kind, we note that we are limited to a single year of data, and multiple factors can change with time to affect cattle movement patterns (e.g., drought, fuel prices, and feed prices). However, we are encouraged that, in addition to the similarities to trends in the U.S. cattle industry noted above, large scale patterns (i.e., state-to-state cattle flows) are similar between summary ICVI data from 2000–2001 [16] and our sampled ICVI data (Figure 1). Thus, despite the potential for yearly variation, our sampled ICVI data are at least good qualitative indicators of the major cattle movement patterns that appear robust to such variation.

**Figure 2:**
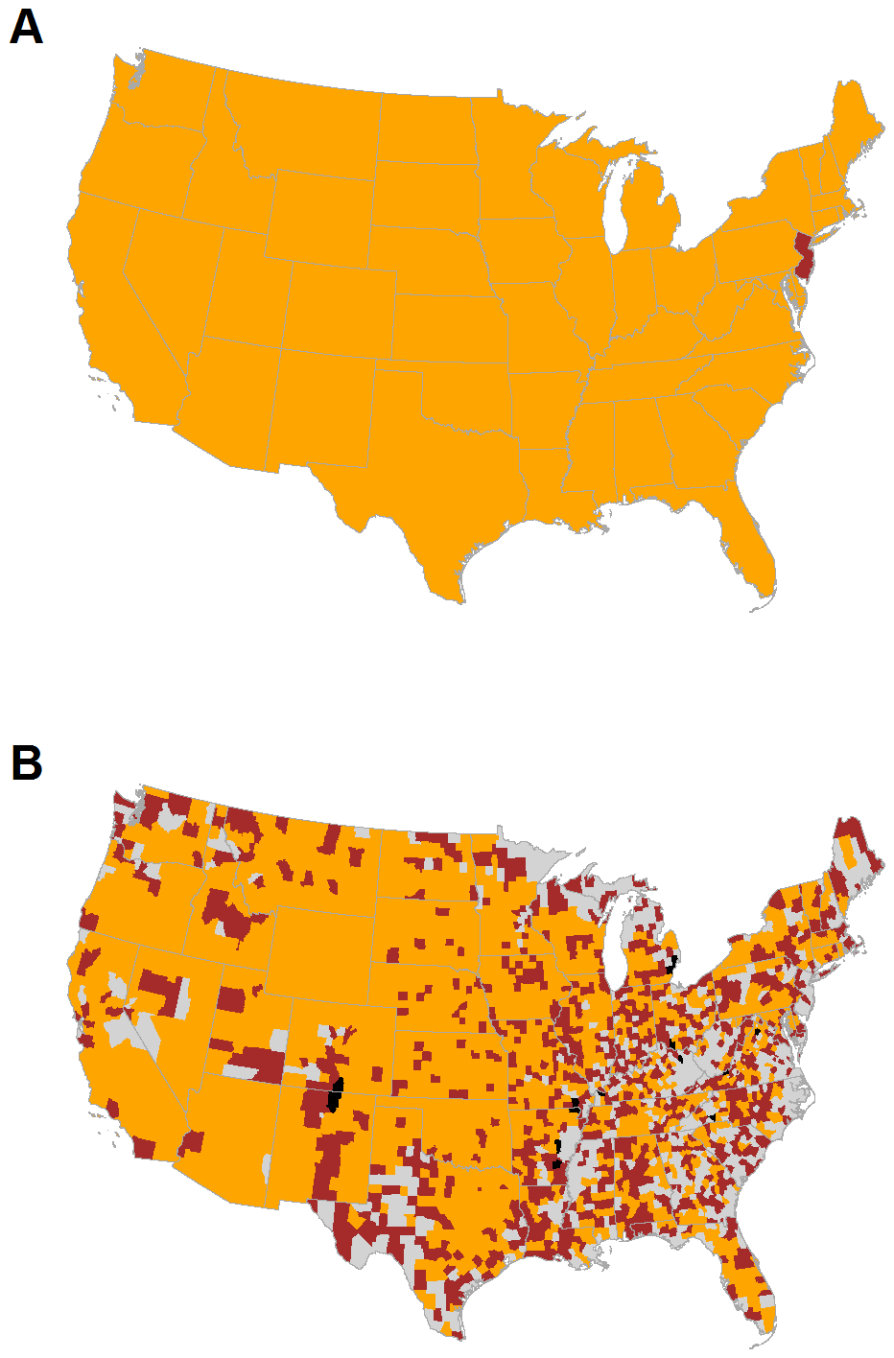
The giant strongly connected component (GSCC) of the network from a 10% sample of ICVIs. Maps at the (A) state and (B) county scales. Orange denotes a node in the GSCC. Brown denotes a node outside of the GSCC that either sends to or receives from nodes in the GSCC but not both, and black indicates nodes that are isolated from the GSCC. Gray indicates no data. New Jersey is outside the state level GSCC because it was the only state not to supply ICVI data.

**Figure 3:**
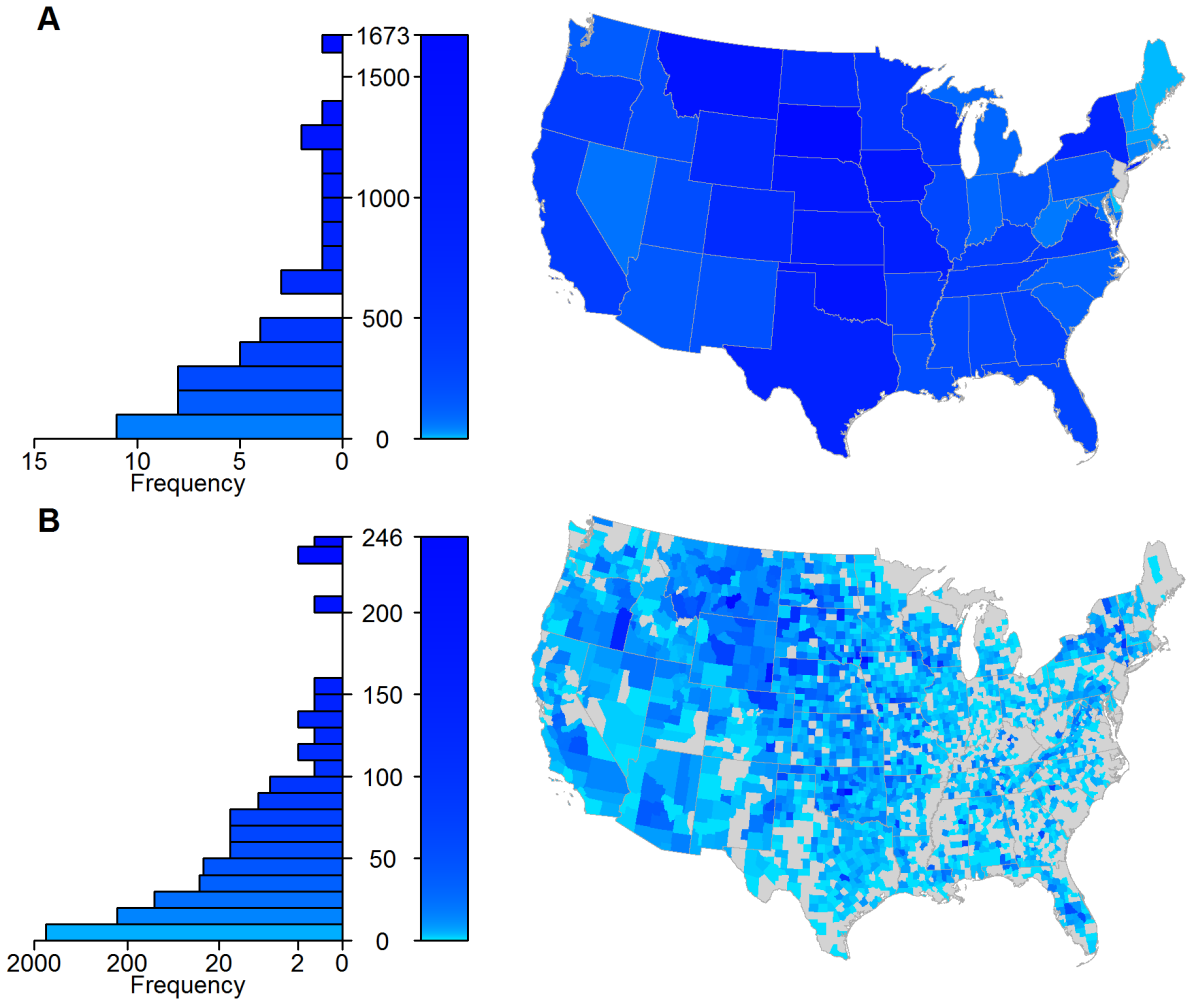
Out-degree distributions of the cattle movement network from a 10% sample of ICVIs. The network is aggregated into (A) state and (B) county nodes. The left-hand graphs show the frequency distribution of node out-degrees, while the maps show the value for that area. A logarithmic color scale is used to differentiate high (dark blue) from low (light blue) out-degree. Counties with no sampled out-shipments are indicated in gray.

**Figure 4:**
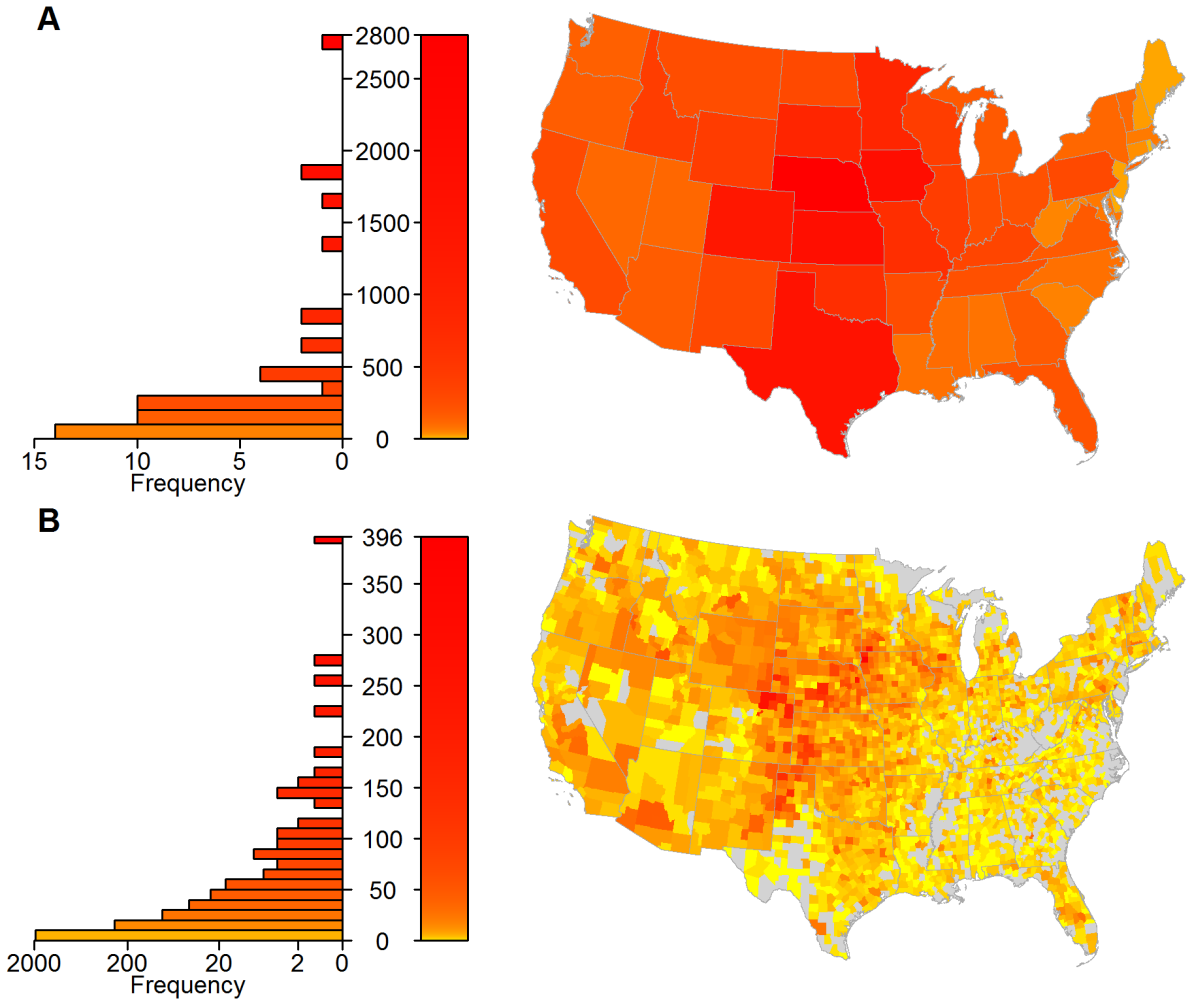
In-degree distributions of the cattle movement network from a 10% sample of ICVIs. The network is aggregated into (A) state and (B) county nodes. The left-hand graphs show the frequency distribution of node in-degrees, whilst the maps show the value for that area. A logarithmic color scale is used to differentiate high (red) from low (yellow) in-degree. Counties with no sampled in-shipments are indicated in gray.

To characterize these patterns and consider spatial heterogeneity in shipments, we aggregated ICVI data at both the state and county scales to create movement networks, with the number of shipments determining the weight of directed edges between nodes. At the state scale, the cattle network consists almost entirely of one giant strongly connected component (GSCC), with the only exception being New Jersey due to its lack of export data (Figure 2A). This GSCC results in a network with a relatively small linear size (i.e., a diameter of 3), potentially allowing cattle, and hence infection, to move between states in a small number of steps. Several geographically central states show higher import and export activity in the cattle movement network (Figures 3A and 4A). At the county scale, the GSCC contains 1551 of the 2433 counties in the network, with other counties being either isolated or only connected in one direction (i.e., by imports or exports but not both) to the GSCC (Figure 2B); in addition, there is a substantial increase in the network distance between nodes (i.e., a diameter of 12). At the county level, import and export activity centers are shifted spatially and exist both within and outside of their state-level counterparts (Figures 3B and 4B). As such, the state scale network aggregates over heterogeneities that are potentially important for disease spread and targeted disease surveillance and control [32].

Owing to the resolution of the available data and the heterogeneities present, we suggest that epidemics are more effectively studied at the county scale. Our ICVI data are a sample of interstate movements, but the data contained numerous short-distance interstate movements. We therefore extrapolate this data to inform the full pattern of movements using a heterogeneous spatial kernel and Bayesian inference methods to generate complete movement networks, including within-state movements [24]. Rather than simulating disease with past movement patterns to determine the spread of infection [10]–[12], [33], we use replicated Bayesian estimates of complete movement networks [24] (i.e., scaling up to all cattle shipments including within-state movements) to explore uncertainty in movement patterns (see Sections C and D in Text S1).

### Metapopulation Disease Model

**Figure 5:**
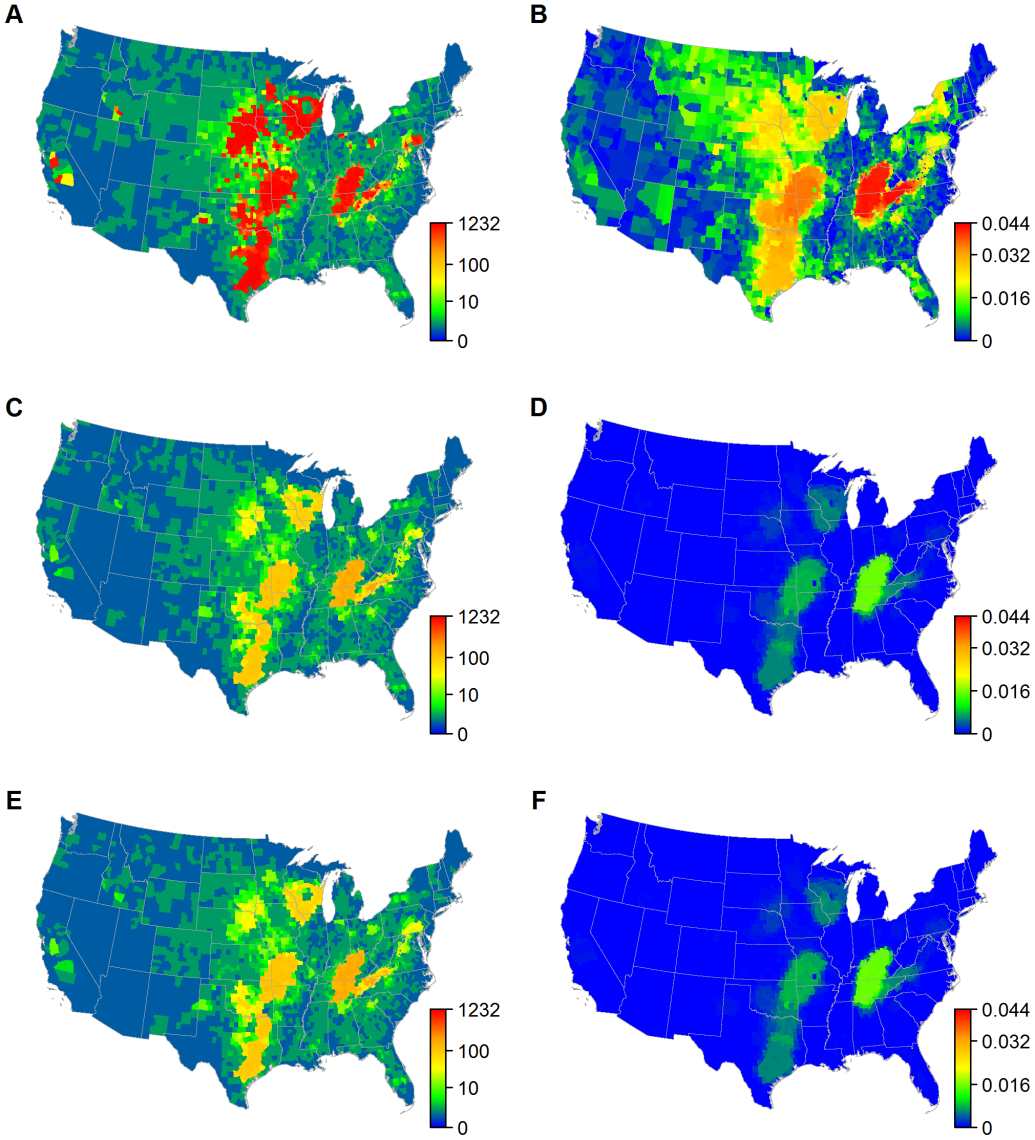
Epidemic extent and infection risk with unrestricted, county and, state movement bans. Upper tail of the distribution (based on the 97.5^th^ percentile of 100 simulations) for epidemic extent and infection risk when infections are introduced to each of the 3109 counties of the continental US. (A & B) assume standard movements while (C & D) assume a county-level movement ban and (E & F) assume a state-level movement ban. (A, C, & E) the epidemic extent (the number of counties infected) for an infection seeded in each county. (B, D, & F) the infection risk (the proportion of all simulated outbreaks that infect a county).

Our model shows that epidemic behavior is strongly dependent on the site of introduction although results are highly stochastic. The largest generated epidemics (i.e., upper 97.5^th^ percentile) are capable of reaching 40% of US counties (the epidemic extent; Figure 5A) and infecting over 120,000 premises (the epidemic size; Figure S2A). When analyzing epidemics, we focus on the upper 97.5^th^ percentile for outbreaks because epidemic extent and size are bimodal: most outbreaks affect 1 or 2 counties (Figure S3A) and less than 10 farms (Figure S2B), but emergency preparedness must address the potential for sustained nationwide epidemics, such as those that arise from the Central Plains and Ohio River Valley in our simulations (Figures 5A and S2A). These regions also experience the greatest risk of infection following introduction elsewhere pointing to potential surveillance and vaccine targets (Figure 5B).

**Figure 6:**
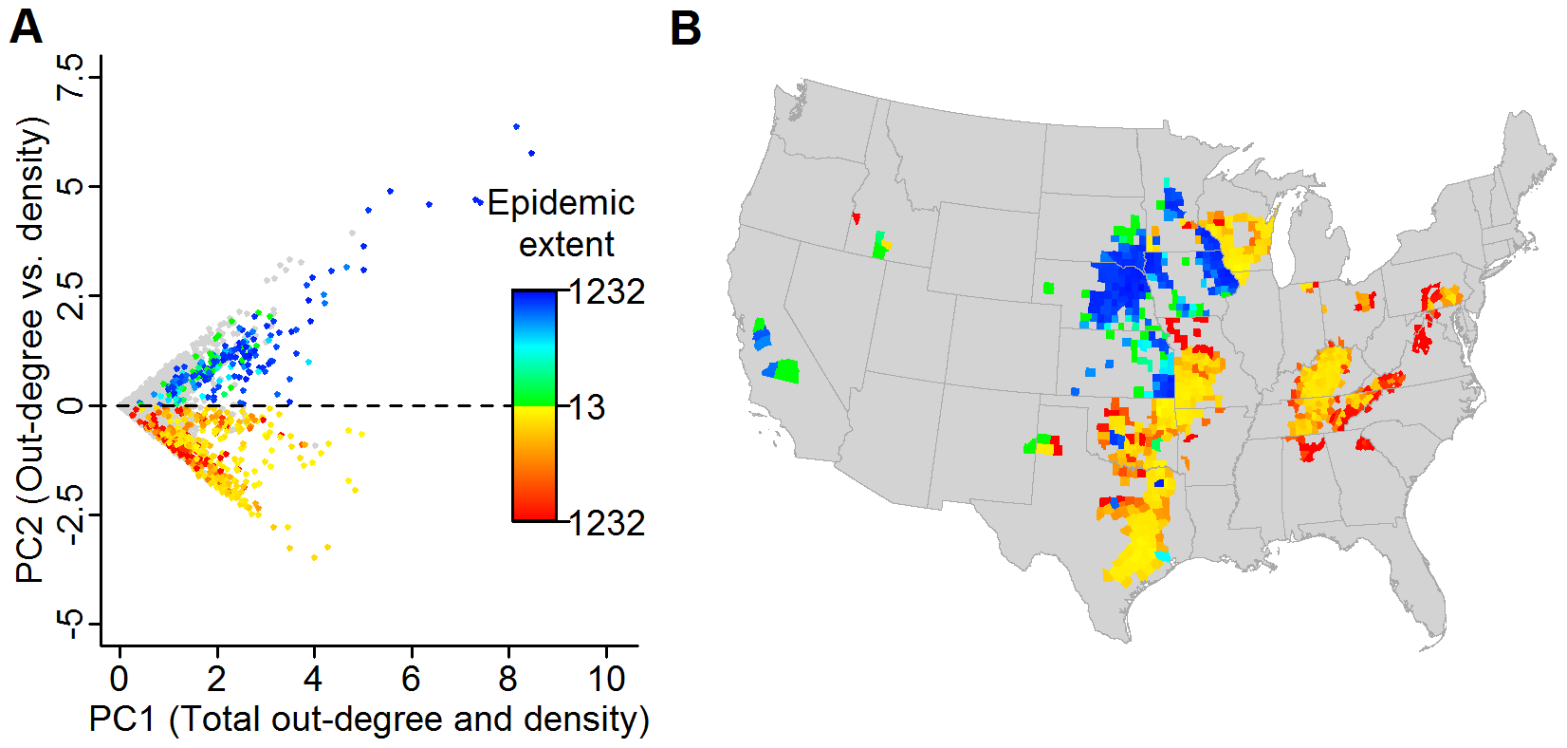
Relative importance of movement vs. local spread determined through a Principal component analysis. (A) Plot of PC1 (0.7071*Out−degree+0.7071*Premises density) vs. PC2 (0.7071*Out−degree+0.7071*Premises density) for each county. Colored dots represent counties in the upper 20% of simulated epidemic extents with the counties where movement is relatively more important (i.e., PC2 > 0) ranging from green to blue and the counties where density is relatively more important (i.e., PC2 < 0) ranging from yellow to red based on epidemic extent. (B) Map depicting the spatial distribution of the counties within the upper 20% of epidemic extents.

With large epidemics spawned from diverse regions of the US, insight for control and surveillance can be gained through an understanding of the heterogeneity in disease spread processes that create the mosaic of outbreak sizes. Because the outputs of our disease simulations were a product of a mixture of local and global processes, simple correlational analyses between a county’s disease outputs and its movements (measured here by the mean out-degree of a county over the 100 predicted networks used in the disease simulations) are confounded by the effect of local spread processes (measured by premises density). To circumvent this problem, we used a principal component analysis on the counties’ out-degrees and premises densities to remove any correlations between the two processes. When we consider the largest epidemic extents (i.e., the counties that generate the largest 20% of uncontrolled epidemic extents denoted by the colored dots in Figure 6A), we see no discernible pattern in the relationship between epidemic extent and these principal components. Spatially, however, we find that counties where movement was relatively more important are found within the clusters of counties that generate large outbreaks (i.e., green to blue regions in Figure 6B). These movement centers are in turn juxtaposed with regions where density is relatively more important (i.e., the orange to red regions in Figure 6B). Thus, local spread processes, here modeled with density-dependence, can result in slow, diffusive spread capable of sustaining itself without long-distance movement but potentially triggering epidemic spread when it reaches a nearby movement hub. Disease spread in the Ohio River Valley appears to be driven almost exclusively due to the impacts of local spread as measured by the effect of cattle density (Figure 6B) requiring potentially different approaches to disease control.

### Controlling Disease Spread with Movement Bans

When infection is detected, cattle shipments from the infected area are likely to be banned to prevent further spread. We focus on movement bans from any county (or state) with known infection. With rapid detection, county-level bans substantially reduce epidemic extent, size and infection risk (Figures 5C-D, S2C-D, and S3C-D) while state bans have little additional benefit (Figures 5E-F, S2E-F, and S3E-F). The sufficiency of county restrictions results from the fragmented distribution of movement centers (Figures 3B and 6B). Local spread away from movement centers is relatively slow in many areas, such that rapid IP removal alone is adequate to prevent the majority of spread across county borders. This means that when infection can be controlled locally, bans beyond the county scale have little additional impact. However, this result will ultimately be modified by the relative influence of local processes on disease spread. Increased density-dependence will decrease the effectiveness of local bans by promoting local, cross-border spread. Thus, the performance of control strategies must be considered in the context of the mechanisms underlying disease spread.

**Figure 7:**
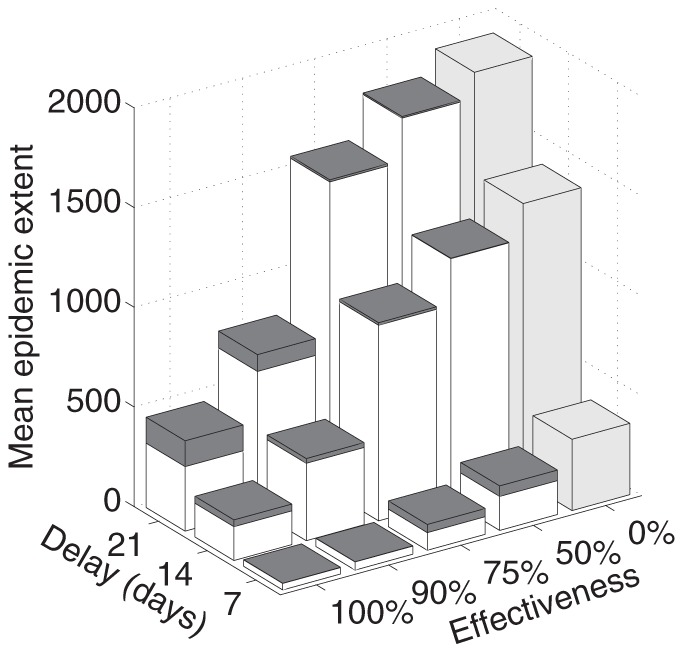
Sensitivity analysis for disease control parameters. Sensitivity of epidemic extent (i.e., number of counties infected) to changes in the delay to implementation and effectiveness of movement bans (i.e., proportion of movements from an area that are stopped). Bars give the mean extent for epidemics begun in the 5% of counties that generate the largest uncontrolled epidemics (as depicted in Figure 5A). The white bars represent a state-level ban while the dark gray bars show the additional epidemic extent if only a county-level ban were introduced. The light gray bars show the no movement ban case.

For the results above, we assumed the delay from a farm becoming infectious to its removal was 7 days (i.e., the infectious period), at which point a 100% effective movement ban was also introduced (i.e., all movements to/from and within the targeted area are prevented). Although this assumption is based on observed detection for the UK [3], [6], it may be optimistic in the US where the scale of the industry may hamper detection and control. Longer delays before IP culling and movement bans increase the epidemic extent dramatically for some source counties (Figures 7 and S4), as these delays allow both a greater degree of local spread and a greater risk of moving infected cattle. Consequently, for a delay of 21 days, a county ban cannot readily contain infection, and a state ban results in marked reductions in epidemic extent (Figure 7). Less effective movement bans (i.e., where a proportion of shipments still occur) result in an increase in the mean epidemic extents due to counties that produce epidemics that ultimately affect over 1000 additional counties, a scale rarely observed under a completely effective ban (Figure S4). As the effectiveness decreases from 100% to 50%, even more differentiation between the state and county bans is observed (Figure 7). We therefore conclude that IP removal and movement control must be introduced rapidly and with reasonable effectiveness for county level control to be sufficient. Any significant delays in detection favor the use of a state ban with an emphasis on ban effectiveness.

## Conclusions

Generalizing kernel-based disease models in UK cattle [3]–[8] to larger cattle systems, such as the United States, has been difficult with insufficient spatial resolution and alignment among oftentimes incomplete data sets to capture inherently complex contact networks. By integrating novel movement data, network scaling advances, and metapopulation disease models that absorb location uncertainties with a flexible kernel-based spread model to explore disease impacts, we illustrate the potential to explore disease spread and control in large, complex, and relatively data-poor systems like the US cattle industry. Our modeling framework advances previous models of cattle disease spread in the US [17]–[19] by using the sampled ICVI data to estimate complete contact networks for the entire country, which is a noted gap in applying previous FMD models, even to regional spatial scales [34], [35]. In addition, parametric distance distributions have been used to describe local transmission processes among individual premises in previous US simulation models spanning county [18] and national scales [17]. Notably, our model represents a trade-off in scale: the coarse data and modeling resolution (relative to individual premises modeling) does not require information on the spatial locations of all cattle premises in the US. Rather, in this study, county-level demographic information is sufficient to characterize disease spread and inform policy at epidemiologically and policy relevant spatial scales.

Yet without a previous significant epidemic, disease models in the US need to be largely informed by outbreak data from countries with cattle industries of different size and structure. Therefore, when faced with an outbreak in the US, rapid estimation of epidemiological parameters is crucial to assess appropriate control measures. Indeed, model sensitivity analyses (see Section E in Text S1) show that levels of infection are strongly parameter dependent (Figure S5A), supporting the need for quick parameterization of models during an outbreak. In contrast to previous U.S. simulation models [17], [19], the relatively parsimonious model structure used in this study facilitates such estimation due to the small number of parameters to be estimated. However, despite the sensitivity of model outputs to specific parameter values, the relative pattern of county-level heterogeneities is robust against parameter variation (Figure S5B). Thus, despite considerable uncertainty in parameter values, spatial patterning in disease impacts is qualitatively, although not necessarily quantitatively, consistent.

However, parameter variation is not the only potential source of uncertainty to be addressed in models of disease spread in the US cattle industry. Recent work has found that daily fluctuations in cattle movement patterns can be an important feature of European network models affecting node centrality and transmission potential in both time and space [36], [37]. Unfortunately, daily networks resulting from our ICVI data are sparse owing to current data constraints. Thus, care must be taken to identify a temporal resolution (e.g., seasonal) that captures actual trends in movement, as opposed to sampling artifacts, in future modeling efforts. In addition, logistical constraints necessarily limited our data collection to cattle ICVIs. However, spread of some livestock diseases (e.g., FMD) may impact species outside of cattle. Future data collection efforts in the US should focus on the potential interaction between livestock industries and in particular, the interaction between long-distance movements and inter-specific local spread.

Despite these potential limitations, our model provides the first truly nationwide assessment of the potential mechanisms, spatial patterns, and impacts of an FMD-like disease outbreak in US cattle. Given the difference in spatial scale between the US and the more well-studied European systems, it is valuable to identify such risk areas for targeted planning and control as we have done here. In particular, the near-continental scale of our model makes state-scale interventions more similar to national-scale interventions in European contexts. We found that more local movement controls, contrary to national or state-scale moratoriums, are often sufficient to control the largest epidemics, although the scale of intervention critically depends on the speed and effectiveness of control. Local movement controls enhance business continuity, a finding with wide appeal for food security, animal welfare, and economic issues not only in the US but also internationally where these local movement controls have not been thoroughly explored. Thus, this modeling framework provides a crucial tool for assessing the efficiency of disease mitigation control measures not only in the US cattle industry, but in numerous data-poor systems where disease spread over large regions is a concern. Future models must continue to explore a wide variety of potential strategies and epidemiological scenarios.

## Supporting Information

Figure S1 **Graphical representation of the spatial variables found in Ω_C_ and Ω_C,C1_ (see Section D in Text S1).**
(TIF)

Figure S2 **Upper tail of and median epidemic size with unrestricted, county, and state movement bans.** Epidemic size (the number of premises infected) when infections are seeded in each of the 3109 counties of mainland USA. (A, C, E) show the upper tail of the distribution (based on the 97.5^th^ percentile of 100 simulations seeded in a county), while (B, D, F) show the median epidemic size (based on the median of 100 simulations seeded in a county) under (A, B) standard movements, (C, D) a county-level movement ban, and (E, F) a state-level movement ban.
(TIFF)

Figure S3 **Median epidemic extent and infection risk under unrestricted, county, and state movement bans.** Median epidemic extents and infection risks (based on the medians of 100 simulations) when infections are seeded in each of the 3109 counties of mainland USA. (A, C, E) show the median epidemic extents (the number of counties infected), while (B, D, F) show the median infection risks under (A, B) no movement ban, (C, D) a county-level movement ban, and (E, F) a state-level movement ban. The bimodality in epidemic behavior is apparent when comparing epidemic extents here to the much larger epidemics seen in Figure 5.
(TIFF)

Figure S4 **Sensitivity of epidemic extent to delay to implementation and effectiveness of a movement ban.** The frequency distributions of epidemic extent for a 7-day (top panel), 14-day (middle panel), and 21-day (bottom panel) delay to the implementation of a county (blue bars) or state (green/yellow bars) movement ban. Ban effectiveness decreases from 100% (county ban – dark blue; state ban – dark green) to 75% (county ban – blue; state ban – light green), and 50% (county ban – light blue; state ban – yellow) of movements stopped. The results for the no movement ban case are shown in red.
(EPS)

Figure S5 **Sensitivity analysis for disease transmission parameters.** Sensitivity analysis results are from the binomial mixed-model describing the mean number of counties infected in the US. (A) Effect sizes for the fixed effects, including main effects of the parameters and all pair-wise interactions, of the transmission parameters. All fixed effects were significantly different from zero (*p* < 0.05), although the main effects had the largest magnitude effect sizes. (B) Variability in the random, county effects on the transmission parameters. Dashed lines indicate zero values.
(TIFF)

Text S1 **Supplementary methods.** Contains sections with descriptions of (A) Interstate Certificate of Veterinary Inspection (ICVI) collection and entry; (B) Premises density and size data; (C) Bayesian kernel model for complete network estimation; (D) Metapopulation disease model; and (E) Sensitivity analysis of disease transmission parameters.
(DOC)
