# Supplementary figures and images for: The Impact of Movements and Animal Density on Continental Scale Cattle Disease Outbreaks in the United States
Source: PLoS One. 2014 Mar 26;9(3):e91724. doi: 10.1371/journal.pone.0091724 (PMC3966763; doi:10.1371/journal.pone.0091724)

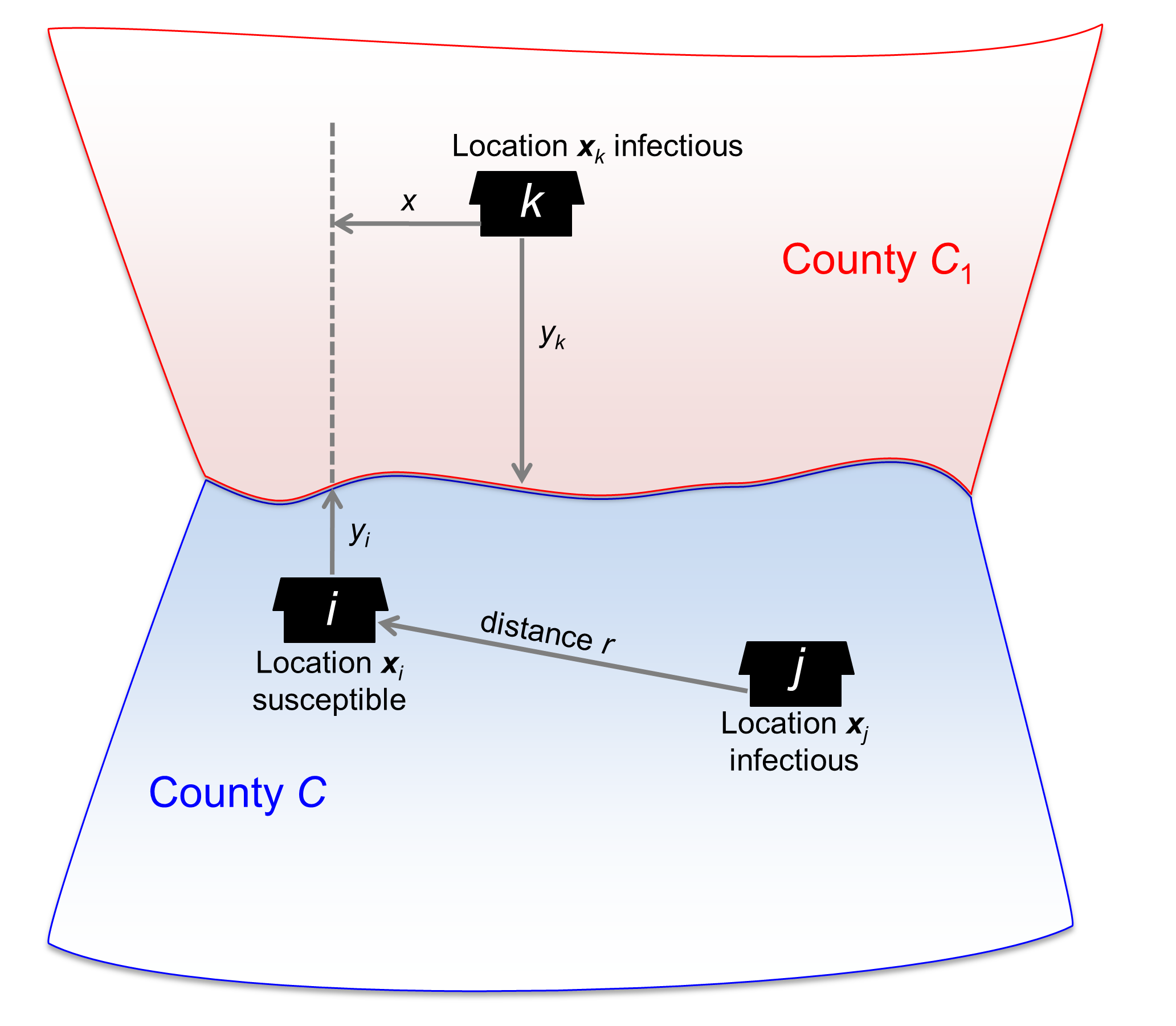

Supplement: Figure S1 — Graphical representation of the spatial variables found in ΩC and ΩC,C1 (see Section D in Text S1). (TIF) [file pone.0091724.s001.tif]

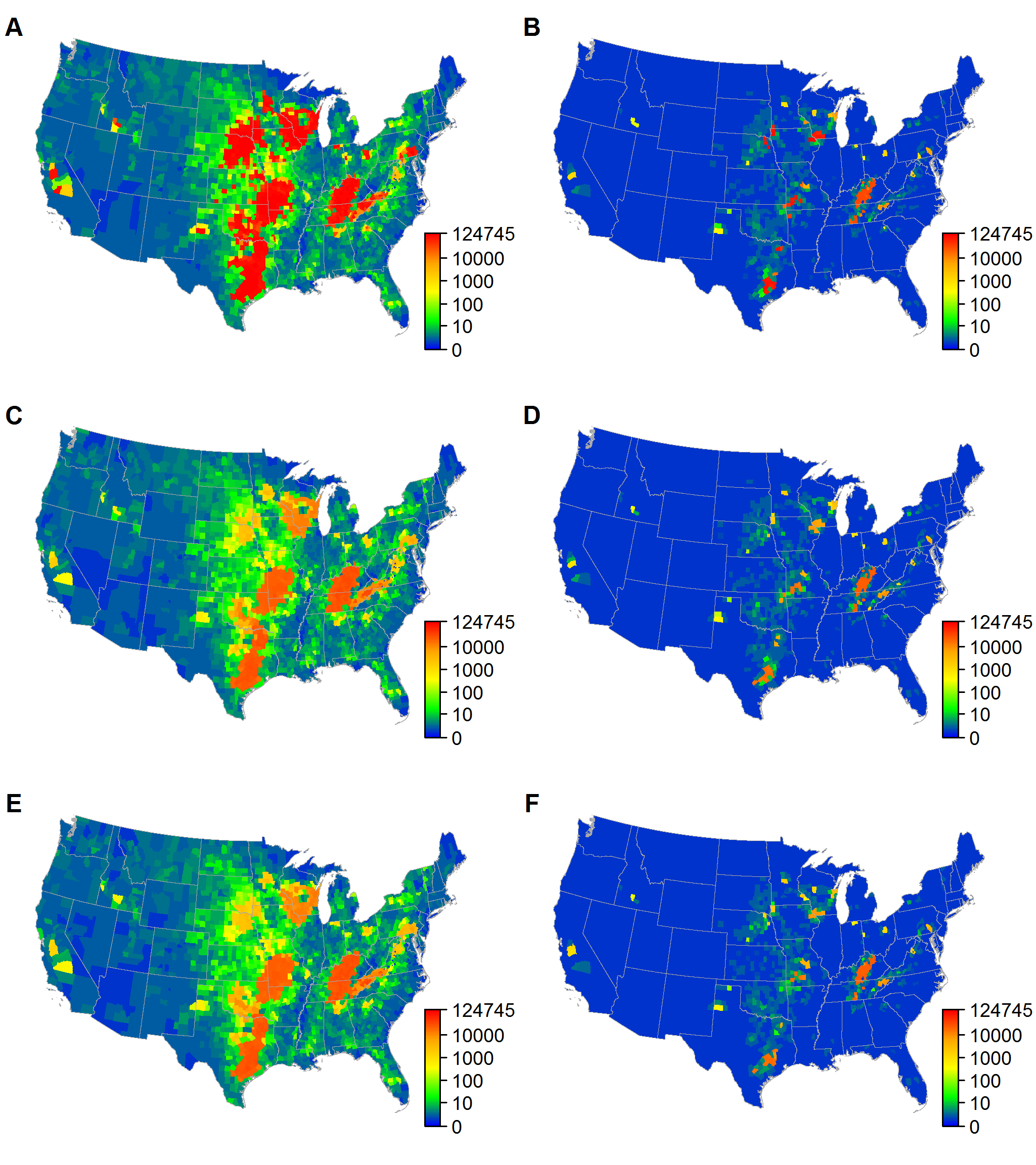

Supplement: Figure S2 — Upper tail of and median epidemic size with unrestricted, county, and state movement bans. Epidemic size (the number of premises infected) when infections are seeded in each of the 3109 counties of mainland USA. (A, C, E) show the upper tail of the distribution (based on the 97.5th percentile of 100 simulations seeded in a county), while (B, D, F) show the median epidemic size (based on the median of 100 simulations seeded in a county) under (A, B) standard movements, (C, D) a county-level movement ban, and (E, F) a state-level movement ban. (TIFF) [file pone.0091724.s002.tiff]

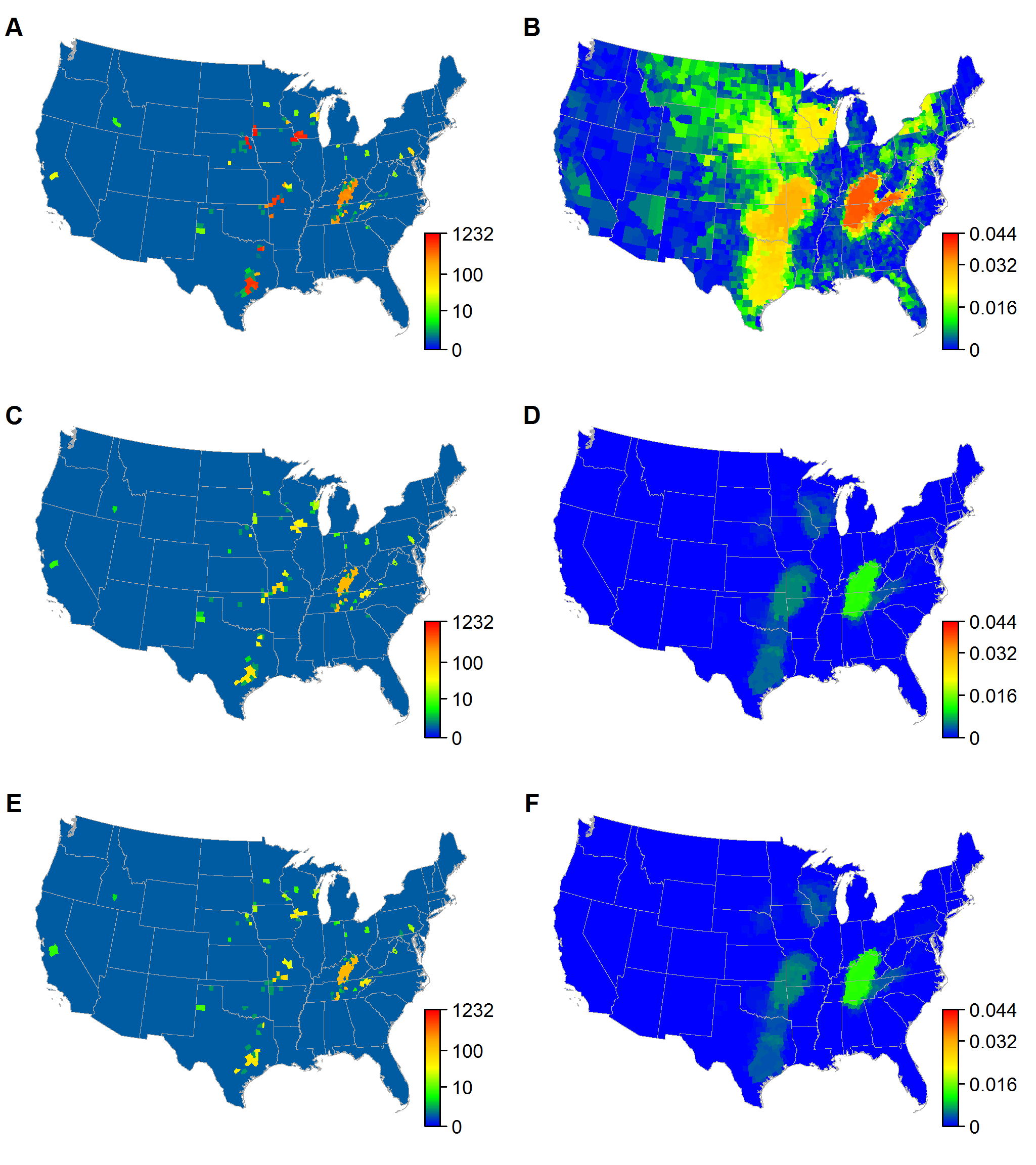

Supplement: Figure S3 — Median epidemic extent and infection risk under unrestricted, county, and state movement bans. Median epidemic extents and infection risks (based on the medians of 100 simulations) when infections are seeded in each of the 3109 counties of mainland USA. (A, C, E) show the median epidemic extents (the number of counties infected), while (B, D, F) show the median infection risks under (A, B) no movement ban, (C, D) a county-level movement ban, and (E, F) a state-level movement ban. The bimodality in epidemic behavior is apparent when comparing epidemic extents here to the much larger epidemics seen in Figure 5. (TIFF) [file pone.0091724.s003.tiff]

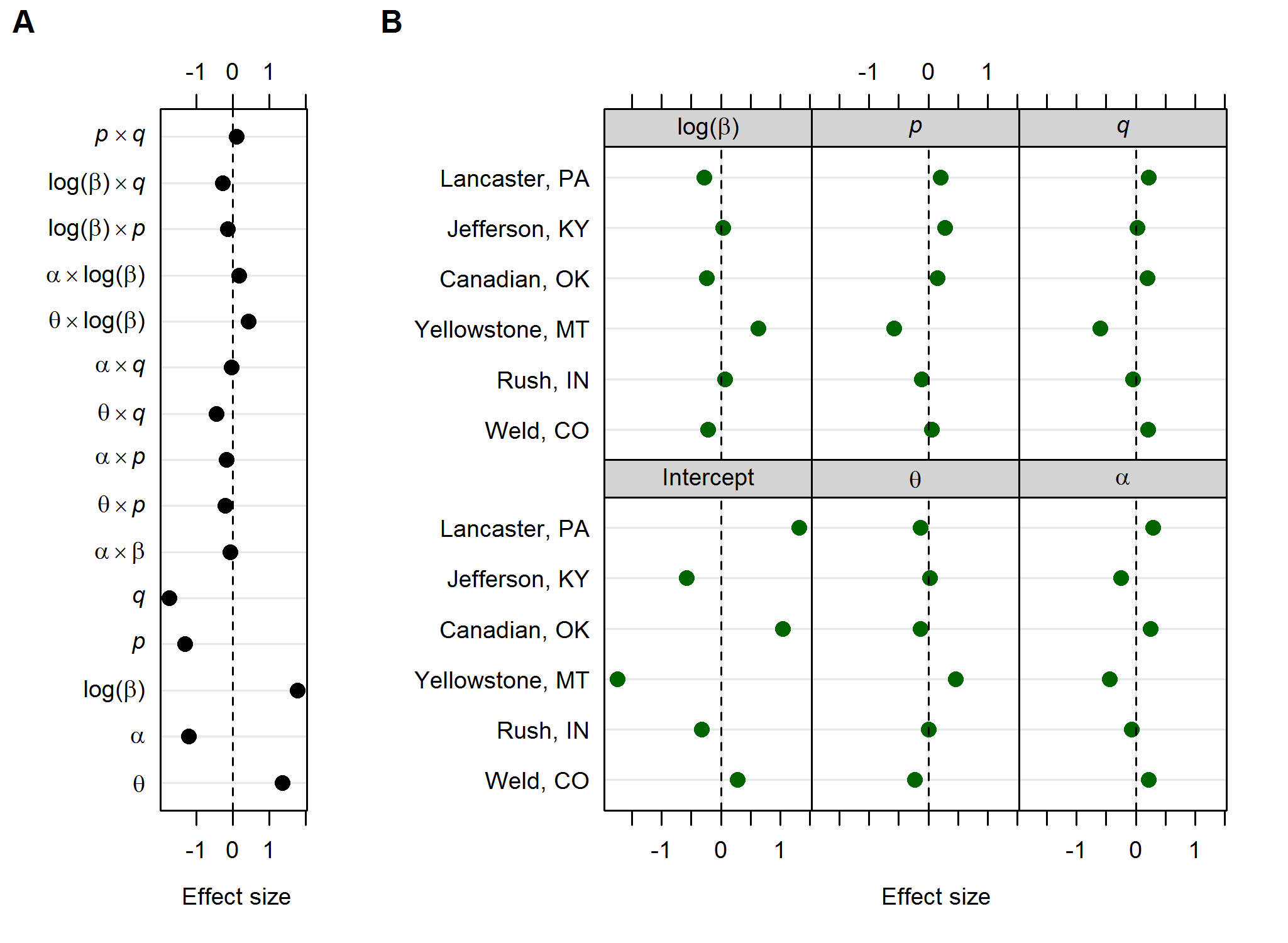

Supplement: Figure S5 — Sensitivity analysis for disease transmission parameters. Sensitivity analysis results are from the binomial mixed-model describing the mean number of counties infected in the US. (A) Effect sizes for the fixed effects, including main effects of the parameters and all pair-wise interactions, of the transmission parameters. All fixed effects were significantly different from zero (p < 0.05), although the main effects had the largest magnitude effect sizes. (B) Variability in the random, county effects on the transmission parameters. Dashed lines indicate zero values. (TIFF) [file pone.0091724.s005.tiff]
