# Supplementary material for: The Impact of Movements and Animal Density on Continental Scale Cattle Disease Outbreaks in the United States
Source: PLoS One. 2014 Mar 26;9(3):e91724. doi: 10.1371/journal.pone.0091724 (PMC3966763; doi:10.1371/journal.pone.0091724)
Supplement: Text S1 — Supplementary methods. Contains sections with descriptions of (A) Interstate Certificate of Veterinary Inspection (ICVI) collection and entry; (B) Premises density and size data; (C) Bayesian kernel model for complete network estimation; (D) Metapopulation disease model; and (E) Sensitivity analysis of disease transmission parameters. (DOC) [file pone.0091724.s006.doc]

**Text S1: Supplementary Methods**

**The Impact of Movements and Animal Density on Continental Scale Cattle Disease Outbreaks in the United States**

Michael G. Buhnerkempe^a,1,2^, Michael J. Tildesley^b^, Tom Lindström^c^, Daniel A. Grear^a^, Katie Portacci^d^, Ryan S. Miller^d^, Jason E. Lombard^d^, Marleen Werkman^b^, Matt J. Keeling^b^, Uno Wennergren^c^, & Colleen T. Webb^a^

*^a^Department of Biology, Colorado State University, Fort Collins, Colorado 80523, USA.*

*^b^Center for Complexity Science, Mathematics Institute, University of Warwick, Coventry, CV4 7AL, UK.*

*^c^Department of Physics, Chemistry, and Biology, Link*öping University, *SE-581 83 Link*öping, Sweden.

*^d^United States Department of Agriculture, Animal and Plant Health Inspection Service, Centers for Epidemiology and Animal Health, Fort Collins, Colorado 80526, USA.*

*^1^Present Address: Department of Ecology and Evolutionary Biology, University of California – Los Angeles, Los Angeles, California 90095, USA; Fogarty International Center, National Institutes of Health, Bethesda, MD 20892, USA*

**Section A:** **Interstate Certificate of Veterinary Inspection (ICVI) collection and entry**

Because ICVIs are housed at state veterinarians’ offices, we requested that each state send us a sample of their ICVIs. To avoid double-sampling an ICVI in one state’s exports and in another’s imports, we requested that the sample be taken from the export ICVIs. Initial contact with state veterinarians’ offices indicated that sampling exports would be minimally burdensome on personnel as import ICVIs are often accompanied by additional documentation. Additionally, ICVIs are oftentimes only stored in the state offices for 1-3 years before being moved to an offsite location. Thus, to facilitate sampling at the time of request in 2010, we asked that states sample 2009 export ICVIs, the most recent full year of data available at the time.

ICVIs were sampled systematically by taking every 10^th^ cattle record. In most cases states either sent the 10% sample or sent all of their 2009 export ICVIs, which were subsequently sampled using the same design. The exceptions to this were:

1. Kentucky – storage of records in county offices made sampling of records difficult. Owing to the fact that there are 40 major markets in Kentucky all located in separate counties, a complete set of records from these counties were obtained to capture a majority of the movements from Kentucky. In addition, a complete set of records from 5-10 of the remaining 80 counties were obtained to characterize movements from these “minor” counties. State officials estimated this sample was approximately 25% of shipment records. We then alternated taking every 2^nd^ and 3^rd^ ICVI to obtain a 10% sample.
2. Missouri – sent an approximate 75% sample. We then alternated taking every 7^th^ and 8^th^ ICVI to obtain a 10% sample.
3. Vermont – sent an approximate 75% sample. We then alternated taking every 7^th^ and 8^th^ ICVI to obtain a 10% sample.

We developed an electronic data entry form tied to a Microsoft Access database for entry of ICVIs. Data fields collected included: origin and destination addresses, shipment date, purpose of shipment, beef/dairy classification, whether the animals were brucellosis vaccinated or TB tested, number of animals, and breed, sex, and age information. Once data were entered, addresses were geocoded. Of the data fields collected, address information at the city level was the most complete with approximately 98% of addresses containing at least a city and state designation. Other fields varied in quality between states [1]. All entered records were double-checked for accuracy and completeness.

**Section B:** **Premises Density and Size Data**

Premises densities within counties were taken from the 2007 Census of Agriculture conducted by the USDA National Agricultural Statistics Service (NASS) as this was the closest census to the 2009 ICVI data [2]. We use “premises” as a general term for any type of premises where cattle are traded as a commodity according to the NASS definition: any establishment from which $1,000 or more of agricultural products were sold or would normally be sold during the year [2].

Premises size distributions (see section D below) were obtained by de-aggregating the county NASS data [2]. We utilize a maximum likelihood approach to fit a high-degree polynomial to the frequency of premises size, such that we obtain the most likely match to the reported number of animals and the number of premises within a particular premises size class within each county. Data on inventory classes that were censored for confidentiality concerns were subsequently filled in to match total county inventory.

**Section C: Bayesian Kernel Model for Complete Network Estimation**

We used the methods described in Lindström et al. [3] to simulate complete cattle movement networks for the US (i.e., all movements including intrastate movements). Briefly, our analysis was based on three different data sets. First, we used the 10% sample of interstate ICVIs. We also obtained the number of premises per county from the National Agricultural Statistics Service (NASS) as described in section B above [2]. Thirdly, in order to incorporate the between state variability in cattle industry infrastructure and production, we used interstate inflow data from 1988-2009 NASS reports of the total number of cattle imported into each state [4].

By Bayesian inference, the width ($V_{S}$, two-dimensional measure of variance) and shape ($K_{S}$, two-dimensional measure of kurtosis) of the kernel describing the decay of the probability of movement with distance as well as total number of shipments ($N_{S}$) for each state, $s$, was estimated jointly. The probability of a destination county, $\delta$, given an origin county, $\omega$, is proportional to$\hat{n}_{i}e^{-\left(D_{\omega, \delta}/a_{S}\right)^{b_{S}}}$, where $D_{\omega, \delta}$ describes the geographic distance between county $\omega$ and$\delta$; $a_{S}$and $b_{S}$are state-specific hyper-parameters governing the kernel distance decay in movement probability; and $\hat{n}_{i}=n_{i}c_{S}$, where $n_{i}$ is the number of premises in county *i* (located in state *s*), and $c_{S}$ is the mean number of animals from the historical inflow into the state. Further, the relationship between$V_{S}$,$K_{S}$ and$a_{S}$,$b_{S}$is given by $V_{S}=a_{S}^{2}\Gamma\left(\frac{4}{b_{S}}\right)/\Gamma\left(\frac{2}{b_{S}}\right)$ and $K_{S}=\Gamma\left(\frac{6}{b_{S}}\right)\Gamma\left(\frac{2}{b_{S}}\right)/\Gamma\left(\frac{4}{b_{S}}\right)^{2}$. As shown in Lindström et al. [3], the behavior of observed, short-distance interstate movements can be used to predict the intrastate movements (and hence the total number of movements) by the assumption that cattle movements are not influenced by state boundaries.

In order to validate our model, we generated networks (i.e., sampled movements from the posterior predictive distribution) comparable to the observed networks as given by the ICVI data. We also generated networks by randomization of the data. We compared relevant network statistics and found that the Bayesian kernel method generally performed well in recapturing the observed structure in terms of mean degree, betweenness, transitivity, reciprocity, and assortativity and that the kernel method substantially outperformed the randomized networks [3]. We also aggregated the networks to the state level and calculated the correlation between predicted and observed networks by a Mantel test. The kernel generated networks showed a correlation of ~0.8, indicating high correlation between the generated and observed structure at this courser spatial scale and also outperformed randomized networks, which showed a correlation of ~0.3 [3].

**Section D:** **Metapopulation Disease Model**

In our model, transmission into county *C* is modeled as a mixture of three processes (Table 1 and Figure S1): local density-dependent within-county transmission (term *W* of Equation S1); local density-dependent transmission from all neighboring counties, $C_{1}$ (i.e., cross-border spread, the *B* term of Equation S1); and transmission from all counties,$C_{2}$, connected to *C* by animal movements (term *M* of Equation S1). With a total number of premises, $F_{C}$, in each county *C* obtained from the NASS data [2], we model the number of premises of each epidemiological classification in each county (Susceptible, $S_{C}$, Exposed, $E_{C}$, Infectious, $I_{C}$, or Removed, $R_{C}$). The stochastic rate at which new infection occurs in county *C* is given by:

| $W_{C}\frac{S_{C}I_{C}}{F_{C}}+\sum_{C_{!}} B_{C,C_{1}}\frac{S_{C}I_{C_{1}}}{F_{C_{1}}}+\sum_{C_{2}} M_{C,C_{2}}\frac{S_{C}I_{C_{2}}}{F_{C_{2}}}$ | (S1) |
| --- | --- |

Description of these terms and the parameters are given below and in Tables 1 and 2, respectively.

We calculate the terms $W_{C}$and $B_{C,C_{1}}$by using a local spatial kernel that implicitly incorporates the impact of distance on contact rates. The kernel is a function of the distance between an infected and susceptible premises and takes a simple but flexible parametric form. The impact of the local spatial kernel is determined by considering the distance between a randomly located susceptible premises and integrating over all possible locations of infected premises in the appropriate counties (see below and Figure S1). This integration is solved numerically by assuming that the counties are large compared to the length-scale of transmission. Movement based transmission ($M_{C,C_{2}}$) is determined by the number of movements between the two counties as determined by realizations of the movement network from the Bayesian model (see section C above). The precise formulation of these three stochastic rates is discussed below.

*Within-County Transmission* ($W_{C}$)

The stochastic rate at which new infections are produced within county *C*, is calculated by initially considering a randomly located pair of premises within the county (premises *i* and *j*). We start by calculating the rate associated with premises *j* infecting premises *i*:

| $\text{Rate}_{i,j,within}=\text{Prob}(i\in S_{C})\times\text{Prob}(j\in I_{C})\times\text{Mean}(Kernel_{i,j}\times Trans_{j}\times Susc_{i})$ | (S2) |
| --- | --- |

where *Kernel*, *Trans* and *Susc* relate to the local spatial kernel (which determines in a spatially implicit manner the neighborhood of influence of an infected premises, i.e., how density-dependent contacts due to aerosol, fence-line contact or fomite transmission changes with distance from the infected premises), the transmission rate from an infected premises and the susceptibility of a susceptible premises, respectively. These latter two terms are assumed to scale non-linearly with the number of animals accounting for both greater viral production and greater than average movement into and out-of larger premises [5-7]. Although we incorporate premises size heterogeneities, we note that production type (e.g., beef vs. dairy) is not incorporated into the local density-dependent transmission. Heterogeneities in premises types, in addition to just premises size heterogeneities, may have an impact on disease dynamics (e.g., local movements of milk trucks), but more data is needed to inform these relationships.

Re-writing the rate equation in terms of known quantities and terms that are assumed independent we achieve:

| $\text{Rate}_{i,j,within}=\frac{S_{C}}{F_{C}}\times\frac{I_{C}}{F_{C}}\times\text{Mean}(Kernel_{i,j})\times\text{Mean}(Trans_{j}\times Susc_{i})$ | (S3) |
| --- | --- |

The two means are then calculated separately as follows:

| $\text{Mean}(Kernel_{i,j})=\int_{C} \int_{C} \frac{1}{A_{C}} \frac{1}{A_{C}}\kappa\left(\left\Vert\mathbf{x}_{i}-\mathbf{x}_{j}\right\Vert\right) d\mathbf{x}_{i} d\mathbf{x}_{j}$ | (S4) |
| --- | --- |

where **x** refers to a vector location of a premises within county *C*, $\kappa\left(r\right)$ is the distance-dependent local spread kernel with *r* describing the distance between any two premises, and the term $1/A_{C}$ (where$A_{C}$ is the area of the county) is used when calculating the probability that a premises is located within an infinitesimal area *d***x**. By assuming that the county is large compared to the scale of transmission, we can merely focus on the distance between two premises:

| $\text{Mean}(Kernel_{i,j})=\frac{1}{A_{C}}\int_{0}^{\infty} 2\pi r\kappa(r)dr=\frac{d_{C}}{F_{C}}\Omega_{C}$ | (S5) |
| --- | --- |

where the number of premises ($F_{C}$), the density of premises ($d_{C}$) and the area of a county ($A_{C}$) are related through the expression$F_{C}=d_{C}\times A_{C}$. Throughout we assume the following functional form for the local spread kernel:

| $\kappa(r)=\frac{\zeta}{1+\left(\frac{r}{\theta}\right)^{\alpha}}$ | (S6) |
| --- | --- |

where *r* is the distance between any two premises (Figure S1), *α* and *θ* are the shape and scale parameters of the local spread kernel respectively, and the normalization constant *ζ* is defined such that:

| $\int_{0}^{\infty} 2\pi r \kappa(r)dr=1$ | (S7) |
| --- | --- |

This form has sufficient flexibility to capture two main features of how local, density-dependent transmission has been observed to decay with distance: a short shoulder where very local transmission remains fairly constant, and a long-range tail of infection that decays with a power-law like behavior [8]. In our formulation, we attribute this to the decay of contacts with distance, which can also be thought of as the neighborhood of influence of infected premises. The normalization that is used ensures that the within-county transmission is independent of the parameters *θ* and *α*, as $\Omega_{C}\equiv1$; these two parameters only play a role in local cross-border transmission. This normalization therefore has the advantage that by changing *θ* and *α* it is easy to scale (and therefore parameterize) local cross-border transmission without affecting the strength of within-county transmission.

We now focus on the mean of the transmission rate multiplied by the susceptibility:

| $\text{Mean}(Trans_{j}\times Susc_{i})=\frac{\beta}{F_{C}(F_{C}-1)}\sum_{i=1}^{F_{C}} \sum_{j\neq i}^{F_{C}} \xi(p)H_{i,C}^{p}\times\xi(q)H_{j,C}^{q}=\left\langle TS\right\rangle_{C}$ | (S8) |
| --- | --- |

where, *β* is the transmissibility of infection and *p* and *q* determine the power-law scaling of the effect of herd sizes in county *C* ($H_{i,C}$; see section B above) on susceptibility and transmissibility, respectively. Previous work for the UK suggests that parameters that account for a sub-linear increase in susceptibility and transmissibility as animal numbers on a premises increase provide a closer fit to historical epidemic data than when these powers are set to unity [5-7]. However, by aggregating premises at the county scale, we are now emphasizing county-level premises size distributions instead of the effect of individual premises sizes on national-scale transmission. Because premises sizes are generally large within the US ($H_{i,C}>>1$), even small changes to the exponents *p* and *q* will have a dramatic impact on the overall transmission rate. To remedy this, we rescale transmissibility and susceptibility by $\xi(p)$and $\xi(q)$, respectively, which are defined such that:

| $\begin{matrix} \sum_{C} \sum_{i=1}^{F_{C}} \xi(p)H_{i,C}^{p}=1 \\ \sum_{C} \sum_{j=1}^{F_{C}} \xi(q)H_{j,C}^{q}=1 \end{matrix}$ | (S9) |
| --- | --- |

Thus, the non-linear effect of herd size has no impact on the average transmission at the national-scale, but county-level differences in premises size distributions will affect the relative scaling of transmissibility and susceptibility. Therefore the parameters *p* and *q* will determine the spatial distribution of infection (at the county scale) and will determine if a high density of cattle (*p* and *q* close to 1) or a high density of premises (*p* and *q* close to 0) is most important.

Putting these calculations together, we find that the total stochastic rate is the sum over all premises *i* and *j*. Therefore:

| $\begin{matrix} W_{C}\frac{S_{C}I_{C}}{F_{C}}=\sum_{i\neq j} Rate_{i,j,within}=\sum_{i\neq j} \frac{S_{C}}{F_{C}}\frac{I_{C}}{F_{C}}\frac{d_{C}}{F_{C}}\left\langle TS\right\rangle_{C}=F_{C}^{2}\left(\frac{S_{C}}{F_{C}}\frac{I_{C}}{F_{C}}\frac{d_{C}}{F_{C}}\left\langle TS\right\rangle_{C}\right) \\ \Rightarrow W_{C}=d_{C}\left\langle TS\right\rangle_{C} \end{matrix}$ | (S10) |
| --- | --- |

providing a simple expression for the within-county transmission rate $W_{C}$ that approximates a premises-to-premises formulation of within-county transmission by using premises density $d_{C}$, and an approximation of local transmission that takes into account the premises size distribution of the county, $\left\langle TS\right\rangle_{C}$.

*Local Cross-Border Transmission* ($B_{C,C_{1}}$)

Because local spread does not stop at the county border, we also include spread from all neighboring counties using the local spread kernel. The rate of local density-dependent cross-border transmission can similarly be calculated by considering a randomly located premises *i* in county *C* and a randomly located premises *k* in adjoining county $C_{1}$. Again, considering the rate at which premises *i* is infected by premises *k* is given by:

| $\text{Rate}_{i,k,cross-border}=\frac{S_{C}}{F_{C}}\times\frac{I_{C_{1}}}{F_{C_{1}}}\times\text{Mean}(Kernel_{i,k})\times\text{Mean}(Trans_{k}\times Susc_{i})$ | (S11) |
| --- | --- |

The calculation of the local mean cross-border spread kernel must take into account vector locations of premises within both counties, but takes the same initial form as the within-county spread:

| $\text{Mean}(Kernel_{i,k})=\int_{C_{1}} \int_{C} \frac{1}{A_{C_{1}}} \frac{1}{A_{C}}\kappa\left(\left\Vert\mathbf{x}_{i}-\mathbf{x}_{k}\right\Vert\right) d\mathbf{x}_{i} d\mathbf{x}_{k}$ | (S12) |
| --- | --- |

where the location of the infecting premises $\mathbf{x}_{k}$ is integrated over the entirety of space occupied by county $C_{1}$. Assuming that the counties are large relative to the scale of transmission, and given that they share a border of length $L_{C,C_{1}}$which is also large relative to the scale of transmission, we can reduce the multiple integrals:

| $\text{Mean}(Kernel_{i,k})=\frac{1}{A_{C}}\frac{1}{A_{C_{1}}}L_{C,C_{1}}\int_{-\infty}^{\infty} \int_{0}^{\infty} \int_{0}^{\infty} \kappa\left(\sqrt{x^{2}+(y_{i}+y_{k})^{2}}\right)dy_{ k}dy_{i} dx = \frac{d_{C}}{F_{C}}\frac{d_{C_{1}}}{F_{C_{1}}}L_{C,C_{1}}\Omega_{C,C_{1}}$ | (S13) |
| --- | --- |

where *x* is taken as the distance between the two premises parallel to their border, whileand $y_{k}$ measure the perpendicular distance between each premises and the border of its respective county (Figure S1). As opposed to within-county transmission, a long-range tail to the local spatial kernel (large *θ* and small *α*) can greatly increase the scale of cross-border transmission. The triple integral $\Omega_{C,C_{1}}$ only needs to be calculated once per simulation and is identical for all pairs of adjoining counties ().

Given that the two premises lie in different counties, the mean of the transmissibility and susceptibility can be calculated independently:

| $\begin{matrix} \text{Mean}(Trans_{k}\times Susc_{i})=\frac{\beta}{F_{C}F_{C_{1}}}\sum_{i=1}^{F_{C}} \sum_{k=1}^{F_{C_{1}}} \xi(p)H_{i,C}^{p}\times\xi(q)H_{k,C_{1}}^{q} \\ =\beta\times\xi(p)MH_{C}(p)\times\xi(q)MH_{C_{1}}(q) \\ =\left\langle TS\right\rangle_{C,C_{1}} \end{matrix}$ | (S14) |
| --- | --- |

where $MH_{C}(p)$is the mean of the herd sizes in county *C* raised to the power *p* (0<*p*<1).

We put together these separate terms to calculate the total rate at which new infectious cases are generated in county *C* due to infection in county $C_{1}$ based on the local cross-border transmission:

| $\begin{matrix} B_{C,C_{1}}\frac{S_{C}I_{C_{1}}}{F_{C_{1}}}=\sum_{i,k} Rate_{i,k,cross-border}=\sum_{i,j} \frac{S_{C}}{F_{C}}\frac{I_{C_{1}}}{F_{C_{1}}}\frac{d_{C}}{F_{C}}\frac{d_{C_{1}}}{F_{C_{1}}}L_{C,C_{1}}\Omega_{C,C_{1}}\left\langle TS\right\rangle_{C,C_{1}} \\ =\frac{S_{C}}{F_{C}}\frac{I_{C_{1}}}{F_{C_{1}}}d_{C}d_{C_{1}}L_{C,C_{1}}\Omega_{C,C_{1}}\left\langle TS\right\rangle_{C,C_{1}} \\ \Rightarrow B_{C,C_{1}}=d_{C_{1}}\frac{L_{C,C_{1}}}{A_{C}} \Omega_{C,C_{1}}\left\langle TS\right\rangle_{C,C_{1}} \end{matrix}$ | (S15) |
| --- | --- |

Hence, the local density-dependent cross-border transmission rate ($B_{C,C_{1}}$) is proportional to the density of premises in county $C_{1}$, the length of the border relative to the area of county *C*, the spatially implicit local spread kernel, the mean transmission rate in county $C_{1}$and the mean susceptibility in county *C*.

*Animal Movement Spread* ($M_{C,C_{2}}$)

The last component of transmission is due to animal movements. We let$M(C,C_{2})$be the number of movements from county $C_{2}$ to county *C* over the entire year, predicted by the Bayesian kernel estimation of U.S. cattle movements as described above. Therefore, $M(C,C_{2})/365$ is the daily probabilistic rate of movement to county *C* from county $C_{2}$. In the event that a movement occurs, we calculate the probability that this is from an infected to a susceptible premises assuming that the source and destination premises are chosen randomly from within each county. This probability is therefore equal to the product of the proportion of total premises in county *C* that are susceptible, $S_{C}/F_{C}$, and the proportion of the total premises in the county $C_{2}$ (where the animal shipment originated) that are infected, $I_{C_{2}}/F_{C_{2}}$. Hence, the total daily rate at which new infection arises in county *C* is given by summing over all counties ($C_{2}$) and the probability of moving a shipment of infected animals to a susceptible premises:

| $\begin{matrix} M_{C,C_{2}}\frac{S_{C}I_{C_{2}}}{F_{C_{2}}}=\frac{M(C,C_{2})}{365}\frac{S_{C}}{F_{C}}\frac{I_{C_{2}}}{F_{C_{2}}} \\ \Rightarrow M_{C,C_{2}}=\frac{M(C,C_{2})}{365 F_{C}} \end{matrix}$ | (S16) |
| --- | --- |

When movement restrictions are imposed, the restrictions at the county (or state) scale are introduced when the first premises in that county (state) is removed (enters the R class). When movement restrictions are assumed to be 100% effective, all movements to and from the county are stopped or all movements to, from and within the state are stopped, for the county and state level ban respectively. Alternative levels of ban effectiveness are applied by reducing the value of $M(C,C_{2})$ associated with each county under the ban. For a thorough sensitivity analysis of the delay and effectiveness of movement restrictions, see section E below.

**Section E:** **Sensitivity Analysis of Disease Transmission Parameters**

There is inherent uncertainty regarding both the disease transmission parameters and the parameters influencing the introduction of control strategies for outbreaks of FMD in the US. In the first instance, we naively assumed that an outbreak of FMD in the US would behave in a similar way (with similar parameters) to the 2001 outbreak in the UK, with the caveat that a model such as the one presented here should ideally be parameterized to fit case reporting data during the course of an actual outbreak. Given this uncertainty in epidemiological parameters for the US, rigorous sensitivity analyses were undertaken to understand how these different sources of uncertainty affect model predictions.

We sought to characterize the effect of uncertainty in disease transmission parameters on model outcomes by exploring a broad region of parameter space, as opposed to simply calculating point derivatives by linearizing around the default parameter values. To accomplish this, we chose a subset of counties that were both important to the US cattle industry and representative of the range of infrastructure and production found within the US on which to explore parameter space. These counties were: Rush County, IN; Canadian County, OK; Weld County, CO; Yellowstone County, MT; Jefferson County, KY; and Lancaster County, PA. We chose the first four counties based on expert elicitation on potentially important counties for movement based disease spread in the US. The last two counties were chosen because of the importance of local density-dependent transmission in shaping epidemics seeded in these counties. Because of constraints due to long model runtime, we used a Latin-Hypercube Sampling (LHS) approach to explore parameter space [9]. Here, parameter uncertainty was characterized using uniform distributions that spanned a relatively broad range of potential values for each parameter (i.e., [2.1, 6] for *α*; [1, 6] for *θ*; [2x10^-5^, 4x10^-2^] for *β*; and [0, 1] for *p* and *q*). We then divided these distributions into 1000 equal-probability intervals and sampled one value from each interval. Parameter sets were constructed by randomly combining the 1000 values for each parameter. Simulations for each parameter set were run on 100 different movement kernels seeded in each of our 6 counties of interest.

We recognize that the local spatial kernel parameters, *α* and *θ*, reflect only sensitivity with regard to their impact on cross-border spread and not on within-county spread. For within-county spread, the current assumptions that premises are randomly distributed and that the county is large compared to the scale of transmission imply that the mean transmission within the county is the same for all values of *α* and *θ*, given the rescaling of the spatial kernel such that the integral $\Omega_{C}=1$. However, this is not the case for cross-border transmission where a long-range tail to the spatial kernel (large *θ* and small *α*) can greatly increase the scale of cross-border transmission. Similarly the herd-size exponents *p* and *q* do not change the average transmissibility and susceptibility across the entire country, but will change how these quantities are distributed. Consequently, both *p* and *q* are expected to have an effect on the overall epidemic extent and size.

To determine sensitivity, we fit a binomial mixed model to the mean number of counties infected as a result of seeding in each of the 6 counties of interest using the ‘lme4’ package [10] in R statistical software [11]. We included a fixed effect for each of the disease transmission parameters (i.e., *α, θ, β, p*, and *q*) as well as all pair-wise interactions of these parameters. We also included county random effects on the intercept as well as slope for each of the disease parameters. This allowed us to not only confirm a single parameter’s importance (measured by the magnitude of the main effects of the parameters) but also to determine how consistent these effects were across the US (measured by the random effect of county) and if any combination of parameter effects (measured by the magnitude of the interaction terms) drastically affects model behavior.

We fit the binomial mixed-effects model to epidemic extent, where the magnitudes of the effects can be used to interpret the relative sensitivities of the parameters. We found that the main effects of all the parameters were all significantly different from zero with large magnitudes (Figure S5A). In contrast, the interaction terms had much smaller magnitude effect sizes, although they were all statistically different from zero (Figure S5A). Thus, interactions between parameters had relatively little impact on model results when compared to the main effects of the disease transmission parameters themselves. Additionally, the main effects of the disease parameters showed relatively little variability across counties (Figure S5B). This indicates that the uncertainty in parameter values affects all counties in a similar way. Thus, we conclude that our results are qualitatively accurate despite the considerable uncertainty.

These sensitivity results were verified using partial-rank correlation coefficients (PRCC) between the parameter values and the number of counties infected [9] that avoid the linear assumptions inherent in the mixed model approach. For all counties, the PRCC values showed the same patterns of effect sizes and directions as in the binomial mixed model.

**Supplementary References**

1. Portacci K, Miller RS, Riggs PD, Buhnerkempe MG, Abrahamsen LM (2013) Assessment of paper Interstate Certificates of Veterinary Inspection to support disease tracing in cattle. J Am Vet Med Assoc 234: 555-560.
2. National Agricultural Statistics Service, US Department of Agriculture (2007) Census of Agriculture. Available: <http://www.nass.usda.gov/census>.
3. Lindström T, [Grear DA](http://www.ncbi.nlm.nih.gov/pubmed?term=Grear DA%5BAuthor%5D&cauthor=true&cauthor_uid=23308223), [Buhnerkempe M](http://www.ncbi.nlm.nih.gov/pubmed?term=Buhnerkempe M%5BAuthor%5D&cauthor=true&cauthor_uid=23308223), [Webb CT](http://www.ncbi.nlm.nih.gov/pubmed?term=Webb CT%5BAuthor%5D&cauthor=true&cauthor_uid=23308223), [Miller RS](http://www.ncbi.nlm.nih.gov/pubmed?term=Miller RS%5BAuthor%5D&cauthor=true&cauthor_uid=23308223), et al. (2013) A Bayesian approach for modeling cattle movements in the United States: scaling up a partially observed network. PLoS ONE 8: e53432. doi: 10.1371/journal.pone.0053432.
4. National Agricultural Statistics Service, US Department of Agriculture (2012) Quick stats Washington, DC. Available: http://quickstats.nass.usda.gov/.
5. Diggle PJ (2006) Spatio-temporal point processes, partial likelihood, foot and mouth disease. Stat Methods Med Res 25: 325-336.
6. Tildesley MJ, [Deardon R](http://www.ncbi.nlm.nih.gov/pubmed?term=Deardon R%5BAuthor%5D&cauthor=true&cauthor_uid=18364313), [Savill NJ](http://www.ncbi.nlm.nih.gov/pubmed?term=Savill NJ%5BAuthor%5D&cauthor=true&cauthor_uid=18364313), [Bessell PR](http://www.ncbi.nlm.nih.gov/pubmed?term=Bessell PR%5BAuthor%5D&cauthor=true&cauthor_uid=18364313), [Brooks SP](http://www.ncbi.nlm.nih.gov/pubmed?term=Brooks SP%5BAuthor%5D&cauthor=true&cauthor_uid=18364313), [et al.](http://www.ncbi.nlm.nih.gov/pubmed?term=Woolhouse ME%5BAuthor%5D&cauthor=true&cauthor_uid=18364313) (2008) Accuracy of models for the 2001 foot-and-mouth epidemic. Proc Biol Sci 275: 1459-1468.
7. Deardon R, Brooks SP, Grenfell BT, Keeling MJ, Tildesley MJ, et al. (2010) Inference for individual-level models of infectious diseases in large populations. Stat Sin 20: 239-261.
8. Keeling MJ, Woolhouse MEJ, Shaw DJ, Matthews L, Chase-Topping M, et al. (2001) Dynamics of the 2001 UK foot and mouth epidemic: stochastic dispersal in a heterogeneous landscape. Science 294: 813-817.
9. Blower SM, Dowlatabadi H (1994) Sensitivity and uncertainty analysis of complex models of disease transmission: an HIV model, as an example. Int Stat Rev 62: 229-243.
10. Bates D, Maechler M, Bolker B (2012) lme4: linear mixed-effects models using S4 classes. Available: <http://CRAN.R-project.org/package=lme4>.
11. R Development Core Team (2012) R: a language and environment for statistical computing. Vienna, Austria: R Foundation for Statistical Computing.
